# Supplementary material for: Trade-offs between sperm viability and immune protein expression in honey bee queens (Apis mellifera)
Source: Commun Biol. 2021 Jan 8;4:48. doi: 10.1038/s42003-020-01586-w (PMC7794525; doi:10.1038/s42003-020-01586-w)
Supplement: Supplementary file 2 — Supplementary Information [file 42003_2020_1586_MOESM2_ESM.pdf]

Supplementary figures: Trade-offs between sperm viability and immune protein expression in honey bee queens (*Apis mellifera*)

Alison McAfee,[1,2*] Abigail Chapman,[2] Leonard J Foster,[2] Jeffery S Pettis,[3] and David R Tarpy[1]

1. Department of Entomology and Plant Pathology, North Carolina State University, Raleigh, North Carolina, USA
2. Department of Biochemistry and Molecular Biology, University of British Columbia, Vancouver, British Columbia, Canada
3. Pettis and Associates LLC, Salisbury, Maryland, USA.

* Corresponding author: alison.n.mcafee@gmail.com

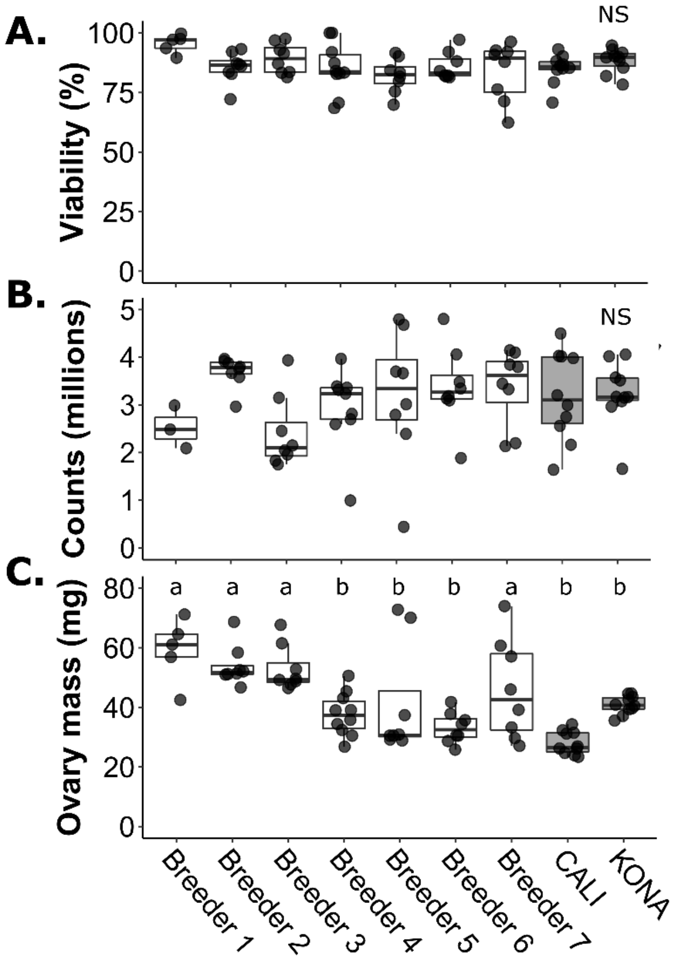

Supplementary Figure 1. Data presented in **Figure 1**, separated by queen source. A) Sperm viability, B) sperm counts, and C) ovary mass metrics for healthy n = 52 healthy queens donated by 7 queen producers within British Columbia, n = 10 queens imported from California, and n = 10 queen imported from Hawaii. No differences in sperm viability nor counts were detected. Ovary masses varied significantly (one-way ANOVA), but this was likely an artifact of differences of caging time between producers. Lowercase letters indicate significant differences ($p < 0.05$). In all cases, boxes represent the bounds between the 2nd and 3rd interquartile range, midlines represent the median, and whiskers are extended by 1.5 times the interquartile range.

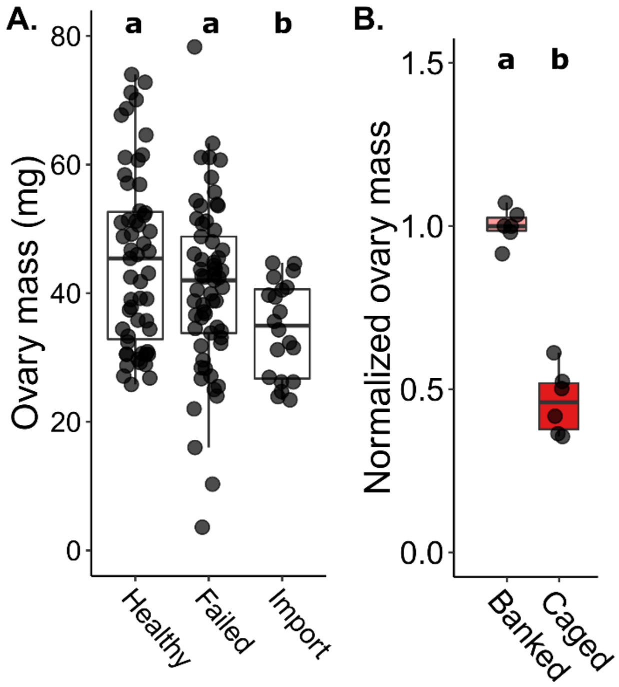

Supplementary Figure 2. Queen ovary masses. A) Ovary mass data from healthy (n = 52), failed (n = 53), and imported (n = 20) queens (data also presented in **Figure 1**). Ovary mass of imported queens was significantly lower than healthy and failed. Lowercase letters indicate significant differences ($p < 0.05$). See **Table 1** for summary statistics. B) Ovary masses from imported Californian queens were either measured upon arrival (‘Caged,’ n = 6) or banked in a queenless colony (‘Banked,’ n = 6) for two weeks prior to measuring ovary mass. Lowercase letters indicate significant differences (t test, $p < 0.05$). In all cases, boxes represent the bounds between the 2nd and 3rd interquartile range, midlines represent the median, and whiskers are extended by 1.5 times the interquartile range.

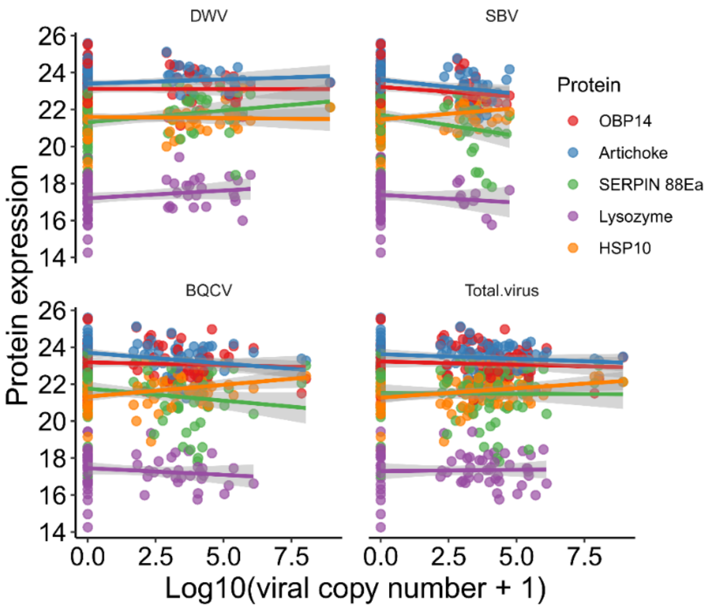

Supplementary Figure 3. No associations between expression of top five proteins and viral factors. N = 94 queens had complete sets of viability, proteomics, and viral data. We evaluated relationships for each protein separately using a least squares linear model, including sperm viability, protein expression, queen status (levels: healthy, failed, imported), and viral copy numbers for deformed wing virus (DWV), sacbrood virus (SBV), black queen cell virus (BQCV), and total load as fixed effects. No significant associations were identified (p > 0.05), except for protein expression with sperm viability, which we already determined previously (p < 0.005). Shaded grey bands represent the 95% confidence interval. Complete statistical summaries for each protein are below.

```
OBP14:
              Estimate Std. Error t value Pr(>|t|)    
(Intercept)  25.681812   0.686255  37.423  < 2e-16 ***
Viability    -0.030919   0.009021  -3.428 0.000936 ***
DWV          -0.001888   0.086668  -0.022 0.982666    
SBV          -0.090281   0.080970  -1.115 0.267958    
BQCV         -0.011570   0.093597  -0.124 0.901907    
Total        -0.009420   0.110389  -0.085 0.932191    
GroupHealthy  0.256250   0.305502   0.839 0.403916    
GroupImport  -0.165068   0.391746  -0.421 0.674539    

Lysozyme:
             Estimate Std. Error t value Pr(>|t|)    
(Intercept)  20.11135    0.77888  25.821  < 2e-16 ***
Viability    -0.04008    0.01031  -3.886 0.000285 ***
DWV          -0.03115    0.10554  -0.295 0.769041    
SBV           0.01943    0.10169   0.191 0.849205    
BQCV         -0.14229    0.11599  -1.227 0.225321    
Total         0.12993    0.13632   0.953 0.344849    
GroupHealthy  0.49760    0.32472   1.532 0.131366    
GroupImport   0.88335    0.46990   1.880 0.065628 .  

HSP10:
              Estimate Std. Error t value Pr(>|t|)    
(Intercept)  19.403701   0.610623  31.777  < 2e-16 ***
Viability     0.024937   0.008026   3.107  0.00256 ** 
DWV          -0.039723   0.077116  -0.515  0.60780    
SBV           0.005754   0.072046   0.080  0.93653    
BQCV          0.059086   0.083282   0.709  0.47995    
```

```
Total          0.078047   0.098223   0.795  0.42904
GroupHealthy -0.253591   0.271832  -0.933  0.35349
GroupImport  -0.337728   0.348572  -0.969  0.33532
```

```
Serpin 88Ea:
             Estimate Std. Error t value Pr(>|t|)
(Intercept)  24.92732    0.88853  28.054  < 2e-16 ***
Viability    -0.04134    0.01168  -3.540 0.000649 ***
DWV           0.04284    0.11221   0.382 0.703604
SBV          -0.17599    0.10484  -1.679 0.096847 .
BQCV         -0.18008    0.12119  -1.486 0.140947
Total         0.11954    0.14293   0.836 0.405252
GroupHealthy -0.13615    0.39555  -0.344 0.731543
GroupImport   0.56905    0.50722   1.122 0.265026
```

```
Artichoke:
              Estimate Std. Error t value Pr(>|t|)
(Intercept)  25.554697   0.554395  46.095  < 2e-16 ***
Viability    -0.024315   0.007287  -3.337  0.00125 **
DWV          -0.006204   0.070015  -0.089  0.92960
SBV          -0.086659   0.065412  -1.325  0.18874
BQCV         -0.144337   0.075613  -1.909  0.05961 .
Total         0.062854   0.089178   0.705  0.48283
GroupHealthy  0.184737   0.246801   0.749  0.45619
GroupImport  -0.042140   0.316474  -0.133  0.89438
```

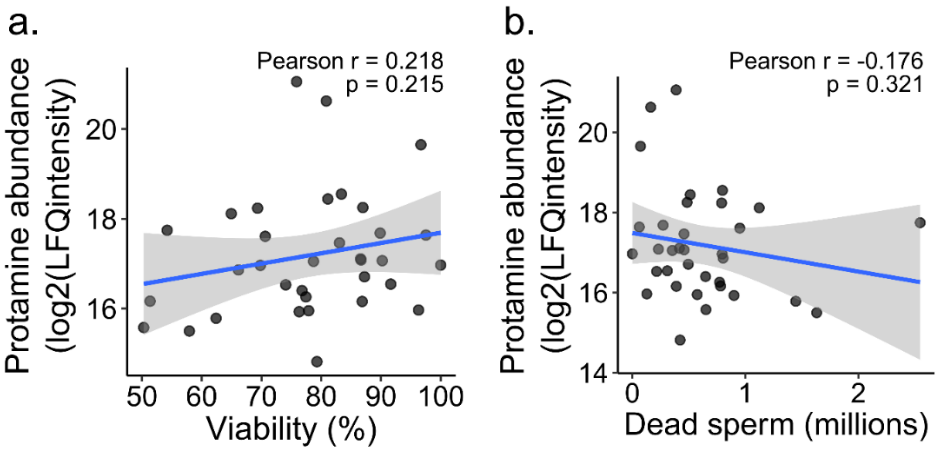

Supplementary Figure 4. Pearson correlations of Protamine-like protein (XP_026294833.1; identified in N = 33 samples) with sperm viability (a) and number of dead sperm (b). Shaded grey bands represent the 95% confidence interval.
