# Supplementary material for: Trade-offs between sperm viability and immune protein expression in honey bee queens (Apis mellifera)
Source: Commun Biol. 2021 Jan 8;4:48. doi: 10.1038/s42003-020-01586-w (PMC7794525; doi:10.1038/s42003-020-01586-w)
Supplement: Supplementary file 3 — Description of Additional Supplementary Items [file 42003_2020_1586_MOESM3_ESM.pdf]

Description of Additional Supplementary Items

File name: Supplementary Data 1 – 7

Description: Excel file containing the supplementary data

Supplementary Data 1: Sample metadata

Supplementary Data 2: Protein Groups (log2)

Supplementary Data 3: Limma output (viability)

Supplementary Data 4: Protein clusters (k = 666)

Supplementary Data 5: Target clusters

Supplementary Data 6: Blast2GO table

Supplementary Data 7: Primers for the 4 targets
