# Supplementary material for: Trade-offs between sperm viability and immune protein expression in honey bee queens (Apis mellifera)
Source: Commun Biol. 2021 Jan 8;4:48. doi: 10.1038/s42003-020-01586-w (PMC7794525; doi:10.1038/s42003-020-01586-w)
Supplement: Supplementary file 5 — Reporting Summary [file 42003_2020_1586_MOESM5_ESM.pdf]

nature research

Corresponding author(s): Alison McAfee

Last updated by author(s): 2020/11/20

Reporting Summary

Nature Research wishes to improve the reproducibility of the work that we publish. This form provides structure for consistency and transparency in reporting. For further information on Nature Research policies, see our Editorial Policies and the Editorial Policy Checklist.

Statistics

For all statistical analyses, confirm that the following items are present in the figure legend, table legend, main text, or Methods section.

n/a	Confirmed	
☐	☒	The exact sample size (n) for each experimental group/condition, given as a discrete number and unit of measurement
☐	☒	A statement on whether measurements were taken from distinct samples or whether the same sample was measured repeatedly
☐	☒	The statistical test(s) used AND whether they are one- or two-sided<br>*Only common tests should be described solely by name; describe more complex techniques in the Methods section.*
☐	☒	A description of all covariates tested
☐	☒	A description of any assumptions or corrections, such as tests of normality and adjustment for multiple comparisons
☐	☒	A full description of the statistical parameters including central tendency (e.g. means) or other basic estimates (e.g. regression coefficient) AND variation (e.g. standard deviation) or associated estimates of uncertainty (e.g. confidence intervals)
☐	☒	For null hypothesis testing, the test statistic (e.g. F, t, r) with confidence intervals, effect sizes, degrees of freedom and P value noted<br>*Give P values as exact values whenever suitable.*
☒	☐	For Bayesian analysis, information on the choice of priors and Markov chain Monte Carlo settings
☐	☒	For hierarchical and complex designs, identification of the appropriate level for tests and full reporting of outcomes
☒	☐	Estimates of effect sizes (e.g. Cohen's d, Pearson's r), indicating how they were calculated

Our web collection on statistics for biologists contains articles on many of the points above.

Software and code

Policy information about availability of computer code

Data collection	ImageJ version 1.52a was used to count sperm cells.
Data analysis	MaxQuant version 1.6.8.0 was used to search the mass spectrometry data. R version 3.5.1 was used to analyze the data. Specific codes are available upon request.

For manuscripts utilizing custom algorithms or software that are central to the research but not yet described in published literature, software must be made available to editors and reviewers. We strongly encourage code deposition in a community repository (e.g. GitHub). See the Nature Research guidelines for submitting code & software for further information.

Data

Policy information about availability of data

All manuscripts must include a data availability statement. This statement should provide the following information, where applicable:

- Accession codes, unique identifiers, or web links for publicly available datasets
- A list of figures that have associated raw data
- A description of any restrictions on data availability

All novel proteomics raw data, search results, and search parameters are available on MassIVE (www.massive.ucsd.edu, accession MSV000085428). Figures associated with these data are Figs 2a, 2b, 2d, 3, 4a-c, and 4f-h. Tables associated with these data are Table 2 and 3. The mass spectrometry data comparing virgins, mated queens, and drone semen has been previously published and is publicly available at www.proteomexchange.org (accession: PXD013728). Sample metadata underlying Fig 1 are available in Supplementary Data S1. Global protein abundances and P values for the correlation between sperm viability and spermatheca protein expression are available in Supplementary Data S2. Hierarchical clustering results of protein-protein correlation coefficients are available in Supplementary Data S4. Any other data that support the findings of this study are available from the corresponding author on request.

Field-specific reporting

Please select the one below that is the best fit for your research. If you are not sure, read the appropriate sections before making your selection.

☒ Life sciences ☐ Behavioural & social sciences ☐ Ecological, evolutionary & environmental sciences

For a reference copy of the document with all sections, see nature.com/documents/nr-reporting-summary-flat.pdf

Life sciences study design

All studies must disclose on these points even when the disclosure is negative.

Sample size	No sample size calculation was performed. The number of queens we analyzed (N = 125 for phenotypic data, N = 123 for proteomics, N = 106 for viral analysis) is the largest queen sample size we are aware of for this type of analysis
Data exclusions	Proteins which were identified in fewer than 10 queens were excluded as these have a higher likelihood of being spurious identifications and are less reliably quantified
Replication	The finding that failed queens have lower sperm viability than healthy queens replicates the findings of Pettis et al. 2016, Plos One. Given the scale of this queen survey, it could not be reasonably replicated with the resources available. We opted to conduct one study with a very large sample size rather than smaller surveys with less statistical power.
Randomization	Queens were assigned to failed and healthy groups based on beekeepers' evaluations. Apiary effects and queen status effects were controlled in the proteomics analysis by including queen status as a fixed effect and apiary as a random variable.
Blinding	Investigators were not blinded. Blinding was not feasible because the main investigator (AM) was in direct contact with collaborating beekeepers and conducted the majority of the laboratory work.

Reporting for specific materials, systems and methods

We require information from authors about some types of materials, experimental systems and methods used in many studies. Here, indicate whether each material, system or method listed is relevant to your study. If you are not sure if a list item applies to your research, read the appropriate section before selecting a response.

Materials & experimental systems

n/a	Involved in the study	
☒	☐	Antibodies
☒	☐	Eukaryotic cell lines
☒	☐	Palaeontology and archaeology
☐	☒	Animals and other organisms
☒	☐	Human research participants
☒	☐	Clinical data
☒	☐	Dual use research of concern

Methods

n/a	Involved in the study	
☒	☐	ChIP-seq
☒	☐	Flow cytometry
☒	☐	MRI-based neuroimaging

Animals and other organisms

Policy information about studies involving animals; ARRIVE guidelines recommended for reporting animal research

Laboratory animals	This study did not involve laboratory animals
Wild animals	This study did not involve wild animals
Field-collected samples	Queens were donated by beekeepers throughout BC, shipped overnight to the laboratory at UBC and analyzed immediately upon arrival, except for imported queens which arrived late at night and were analyzed the following morning. Queens were anesthatized with carbon dioxide prior to decapitation.
Ethics oversight	As non-cephalopod invertebrates, honey bees are not subject to animal ethics approval at UBC.

Note that full information on the approval of the study protocol must also be provided in the manuscript.
